# Real-time monitoring of magnetic drug targeting using fibered confocal fluorescence microscopy

**DOI:** 10.1016/j.jconrel.2016.07.026

**Published:** 2016-12-28

**Authors:** Jie Bai, Julie Tzu-Wen Wang, Kuo-Ching Mei, Wafa T. Al-Jamal, Khuloud T. Al-Jamal

**Affiliations:** a Institute of Pharmaceutical Science, Faculty of Life Sciences & Medicine, King's College London, SE1 9NH, UK; b School of Pharmacy, University of East Anglia, Norwich Research Park, Norwich NR4 7TJ, UK

**Keywords:** Superparamagnetic iron oxide nanoparticles (SPIONs), Magnetic drug targeting, Cellvizio®, Tumor, Nanomedicine

## Abstract

Magnetic drug targeting has been proposed as means of concentrating therapeutic agents at a target site and the success of this approach has been demonstrated in a number of studies. However, the behavior of magnetic carriers in blood vessels and tumor microcirculation still remains unclear. In this work, we utilized polymeric magnetic nanocapsules (*m*-NCs) for magnetic targeting in tumors and dynamically visualized them within blood vessels and tumor tissues before, during and after magnetic field exposure using fibered confocal fluorescence microscopy (FCFM). Our results suggested that the distribution of m-NCs within tumor vasculature changed dramatically, but in a reversible way, upon application and removal of a magnetic field. The m-NCs were concentrated and stayed as clusters near a blood vessel wall when tumors were exposed to a magnetic field but without rupturing the blood vessel. The obtained FCFM images provided *in vivo in situ* microvascular observations of m-NCs upon magnetic targeting with high spatial resolution but minimally invasive surgical procedures. This proof-of-concept descriptive study in mice is envisaged to track and quantify nanoparticles *in vivo* in a non-invasive manner at microscopic resolution.

## Graphical abstract

**Figure:**
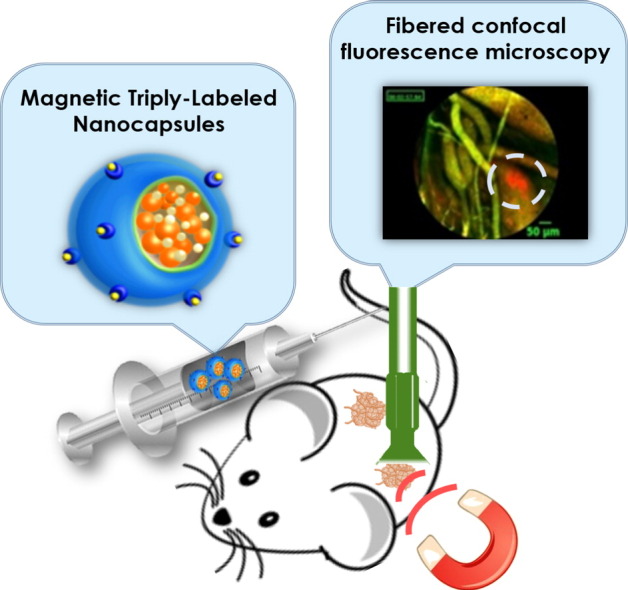

## 1 Introduction

Magnetic drug targeting has been shown to be a promising method of concentrating therapeutic agents at a target site, and allowing higher drug doses to be administered while still being tolerated by patients [1], [2], [3]. A number of studies have demonstrated that magnetic nanoparticles (MNPs) can be magnetically targeted to tumor sites and the targeting effect has been assessed by various techniques, including optical imaging, magnetic resonance imaging (MRI) and histology studies (Prussian blue staining) [4], [5], [6], [7], [8], [9], [10], [11]. These studies have suggested that a higher concentration of magnetic therapeutic agents can be achieved upon the application of an external magnetic field. However, the magnetic behaviors of MNPs in blood vessels and tumor microcirculation have not been investigated. The above mentioned imaging methods cannot offer sufficient resolution to show how MNPs travel through blood vessels and accumulate in tumors, whereas histological studies can only provide *ex vivo* information at post-mortem in a non-dynamic way. Some studies have been carried out using *ex vivo* artery models or mathematical simulation to characterize the MNPs behaviors in blood vessels and/or surrounding tissues, but no *in vivo* studies have been performed so far [12], [13], [14], [15].

Here we visualized the magnetic capture of magnetic polymeric nanocapsules (*m*-NCs) within blood vessels and tumor tissues in real-time *in vivo* before, during and after magnetic field exposure. The magnetic targeting efficacy of m-NCs was firstly quantified by gamma counting and this was further confirmed by direct imaging of the magnetic targeting process in blood vessel on the microscopic scale. This proof-of-concept descriptive study in mice is envisaged to track and quantify nanoparticles *in vivo* in a non-invasive manner with microscopic resolution.

## 2 Materials and methods

### 2.1 Materials

‘Ferrofluid’ magnetic oil (oleic acid-coated superparamagnetic iron oxide nanoparticles (SPIONs) with diameter of 10 nm, suspended in kerosene at 10^17^ particles per mL) was purchased from Magnacol Ltd. (UK). Soybean lecithin (Epikuron 140 V) was a kind gift from Cargill Pharmaceuticals (USA). Polyoxyethylene-bis-amine (NH_2_-PEG_3.5 kDa_-NH_2_) was purchased from JENKEM (USA). d/l-lactide/glycolide copolymer 75/25 (PLGA_18_ _kDa_-COOH) was purchased from Purac Biomaterials (the Netherlands). Tween® 80, nitric acid, methanol, dimethylsulphoxide (DMSO) and dichloromethane were obtained from Fisher Scientific Ltd. (UK). Fluorescein isothiocyanate–dextran (FITC-Dextran, average molecular weight 2000 kDa), sodium chloride, diethylene triamine pentaacetic acid (DTPA), castor oil, ethylenediaminetetraacetic acid disodium salt dehydrate (EDTA) and Sephadex® G-75 were purchased from Sigma Aldrich (UK). 1,1-dioctadecyltetramethyl indotricarbocyanine Iodide (DiR) was obtained from Cambridge Bioscience (UK). Advanced RPMI-1640 media, penicillin-streptomycin 100 ×, 0.25% Trypsin-EDTA with phenol red, Glutamax™ supplement, phosphate buffered saline PBS (10 ×, pH 7.4) and phosphate buffered saline PBS (1 ×, pH 7.4) were obtained from Gibco, Invitrogen (UK). Fetal bovine serum (FBS) was obtained from First-Link Ltd. (UK). Pentobarbital sodium (Euthatal®) was obtained from Merial (UK). PD-10 desalting column was obtained from GE Healthcare Life Sciences (UK).

### 2.2 Preparation and characterization of m-NCs and m-NC-DiR

The magnetic polymeric nanocapsules (*m-*NCs) were prepared by single emulsification/solvent evaporation method [1]. PLGA_18_ _kDa_-PEG_3.5_ _kDa_-NH_2_ and PLGA_18_ _kDa_-PEG_3.5_ _kDa_-DTPA were synthesized as described in our previous work [16], [17]. Briefly, PLGA_18_ _kDa_-PEG_3.5_ _kDa_-NH_2_ (12.5 mg, 10% w/w PLGA_18_ _kDa_-PEG_3.5_ _kDa_-DTPA was incorporated for m-NCs to be used for radiolabeling), castor oil (75 mg), soybean lecithin (25 mg) and increasing amounts of SPIONs (0, 0.5, 2.5 or 10 mg) were dissolved in 2.5 mL dichloromethane. DiR was incorporated into m-NCs formulation at 0.5% w/w DiR/castor oil for fibered confocal fluorescence microscopic (FCFM) imaging. The organic phase was poured into an aqueous phase (5 mL) containing Tween® 80 (20 mg) as a hydrophilic surfactant. The resultant dispersion was emulsified by ultra-sonication using a probe sonicator (Soniprep 150, UK) at 15 μ amplitude for 180 s in an ice bath. Organic solvents were then evaporated in a chemical fume hood for 20 min. The final volume of the m*-*NC suspension was adjusted to 5 mL. The obtained *m*-NC suspension was concentrated using a rotary evaporator (Buchi, Switzerland) by 10 (FCFM imaging) or 20 times (gamma counting), yielding 25 and 50 mg/ml of polymer, respectively.

### 2.3 Size and zeta potential measurements

The hydrodynamic size (*Z*-Average), polydispersity index (PDI) and zeta potential of NCs and m*-*NCs were determined by NanoZS (Malvern Instrument, UK) at 25 °C using disposable square polystyrene cuvettes (for size and PDI) or disposable capillary cells (for zeta potential) (Malvern Instrument, UK). The *Z*-Average diameter and polydispersity index were measured in water and presented as the average value of three measurements, with 15 runs within each measurement. The zeta potential was also measured in water and presented as the average value of three measurements, with 20–25 runs within each measurement. The mean and standard deviation (SD) of size and zeta potential were calculated for each sample.

### 2.4 Determination of SPION encapsulation efficiency in m-NCs

m*-*NCs were prepared with increasing loadings of SPIONs (0, 0.38, 1.84 and 7.02% w/w SPION/NC) and purified by size exclusion chromatography (Sephadex® G-75 column, size exclusion chromatography) to remove any un-encapsulated SPIONs. The Fe content was determined by inductively couple plasma mass spectrometry (ICP-MS, Perkin Elmer SCIEX ICP mass spectrometer, ELAN DRC 6100, USA). For ICP-MS measurements, Fe standards (Leeman Labs Inc., USA) were prepared in 20% nitric acid to obtain a standard curve in the range of 10–10,000 parts per billion with respect to Fe. m*-*NCs were digested in 2 mL of nitric acid in Falcon™ 15 mL conical centrifuge tubes (Fisher Scientific, UK) and incubated overnight at 50 °C. The resulting solution was diluted by 10 times with water before the measurements.

### 2.5 Determination of DiR encapsulation efficiency in m-NCs

The encapsulation efficiency of DiR in m-NCs was assessed using a UV/fluorescence spectrometer (Varian, Cary Eclipse, Australia). Prior to quantification, m-NC-DiR suspensions were purified by a PD-10 desalting column (size exclusion chromatography) and eluted in PBS buffer to remove any free DiR. The m-NC suspensions before and after purification were diluted in DMSO (1/19, v/v) to rupture the NC structure. The excitation/emission wavelengths for the detection of DiR were 740/785 nm. The encapsulation efficiency was expressed as the percentage of the encapsulated dye to the total amount of DiR added to the formulation. All measurements were performed in triplicate and expressed as mean ± SD (*n* = 3).

### 2.6 Radio-labelling of m-NCs and serum stability studies

To radio-label the m*-*NCs with indium-111, m*-*NCs were prepared as described in the previous section except that PLGA_18_ _kDa_-PEG_3.5_ _kDa_-DTPA was included at 10% (w/w) of the total polymer content. The m-NC suspension (250 μL, 50 mg/mL of polymer) was incubated with 2 M ammonium acetate (one ninth of the reaction volume, pH 5.5), to which 1 MBq of ^111^InCl_3_ (Mallinckrodt, UK) was added for gamma counting. The reaction was kept at room temperature for 30 min with intermittent vortexing every 10 min. Upon completion, the radio-labelling reaction was quenched by the addition of 0.1 M EDTA chelating solution (one twentieth of the reaction volume). ^111^InCl_3_ alone was subjected to the same labelling reaction conditions and used as a control.

The m*-*NC-^111^In was passed through PD-10 columns before injecting into animals to exchange the ammonium acetate buffer (pH 5.5) with PBS (pH 7.4) and remove free ^111^In-EDTA. The m*-*NCs-^111^In (~ 150 μL per injection dose, 25 mg/mL of polymer) were collected from the column and spotted on instant thin layer chromatography (iTLC) strips which were then developed in 0.1 M ammonium acetate containing 50 mM EDTA as a mobile phase. Strips were allowed to dry before being developed and counted quantitatively using a cyclone phosphor detector (Packard Biosciences, UK) to ensure no free ^111^In-EDTA present in the injected solution.

### 2.7 Animal studies and tumor inoculation

All animal experiments were performed in compliance with the UK Home Office (1989) Code of Practice for the Housing and Care of Animals used in Scientific Procedures. CT26 murine colon carcinoma cells (CT26, ATCC®, CRL-2638TM) were cultured in Advanced RPMI (Roswell Park Memorial Institute) 1640 medium supplemented with 1% l-glutamine, 1% penicillin-streptomycin and 10% fetal bovine serum (FBS), in 5% CO_2_ and 95% air, at 37 °C. The harvested CT26 cells were suspended in PBS solution (pH 7.4). A total of 1 × 10^6^ cells in 20 μL were injected subcutaneously and bifocally at the hind foot of female BALB/c mice aged 4–6 weeks (Harlan, UK). After inoculation, the tumor volume was measured on day 8 and then every other day using a digital caliper and calculated using Eq. (1) [2]

(1) $$\text{Tumor volume}\;\left({\mathrm{mm}}^{3}\right)=\left(4/3\right)*\pi \;{\left(A/2\right)}^{2}*\left(B/2\right)=0.52A^{2}B$$

where *A* and *B* represent the width and the length of the tumors, respectively. All experiment were carried out (m-NCs administration) when the tumor volume reached approximately 500 mm^3^.

### 2.8 Magnetic targeting setup *in vivo*

Disk-shaped nickel-coated neodymium iron boron (Nd_2_Fe_14_B) magnets (Magnet Expert Ltd., Tuxford, UK) were used for the *in vivo* magnetic drug targeting studies. That was an 8 mm diameter, 5 mm thick, N42 grade magnet (product code F324), which had a reported field strength of 0.43 Tesla (T) and a reported ‘vertical pull’ parameter (a measure of the mass of material that the magnet could lift) of 1.9 kg. Single magnet was placed non-invasively over the surface of one of the bifocal tumors immediately after m-NCs administration and retained using surgical tapes. The contralateral tumor was used as an internal negative control where no magnet was applied. The magnet was then removed at 1 h post-injection of m-NCs.

### 2.9 Fibered confocal fluorescence microscopic imaging studies

The visualisation of m-NCs in tumor vasculature *in vivo* upon the application of a magnetic field was performed using a fibered confocal fluorescence microscopy (FCFM) imaging system (Cellvizio®, Mauna Kea Technologies, Paris, France). CT26 tumor-bearing BALB/c mice were injected *via* a tail vein with m-NC-DiR with a SPION content of 125 mg/kg and DiR content of 9.4 mg/kg (corresponding to 312.5 mg polymer/kg). FITC-Dextran (MW 2000 kDa, 500 mg/kg, 150 μL) was injected intravenously immediately prior to FCFM imaging to visualize the microvascular network. A small incision (~ 0.5 cm) was made on tumor and muscle to insert the laser probe. The exposed tissues were kept moist with PBS pre-warmed to 37 °C. During the time of acquisition, mice were placed in the prone position (for tumor and muscle imaging) or supine position (for liver imaging) and anesthetized with 1.5% isoflurane/98.5% oxygen. Body temperature was controlled by a probe-coupled heating mat. Images and videos were acquired using an S-1500 probe with a penetration depth of 15 μm below tissue surface. To image m-NCs in the tumor vasculature upon exposure to a magnetic field in real-time, a set of magnets (10 disk magnets, 0.43 T, product code F324, Magnet Expert Ltd., Tuxford, UK) was placed adjacent to the surgical incision for 10 min. FCFM imaging was performed before, during and after application of a magnetic field. Extravasation of the m-NCs through blood vessel wall and accumulation over time were also assessed in tumor (TU + and TU −), quadriceps femoris muscle and liver at 1, 4 and 24 h post-injection. All imaging was carried out using a frame rate of 9 Hz (full FOV), a field of view of 618 × 609 μm and 100% laser power at 488 and 660 nm. Images and videos were analyzed using Cellvizio® dual viewer (Mauna Kea Technologies, Paris, France) and videos were reconstructed using ImageJ software. The fluorescence intensity of the signals from DiR and FITC was quantified using ImageJ software.

### 2.10 Assessment of blood circulation, organ biodistribution and tumor accumulation profiles of m-NCs by gamma counting

Blood circulation, organ biodistribution and tumor accumulation profiles of m-NC-^111^In with increasing SPION contents (0, 5, 25 and 125 and 500 mg/kg), referred to as NC 1, NC 2, NC 3, NC 4 and NC 5, respectively, were assessed quantitatively in CT26 tumor-bearing BALB/c mice using gamma counting. Mice were injected intravenously *via* tail vein with ~ 0.7 MBq m-NC-^111^In (150 μL in PBS). Magnetic targeting was applied as described in 2.8. Blood samples (5 μL) were collected from the tail vein at 10 min, 30 min, 1 h, 4 h and 24 h post-injection. Liver, spleen, magnetically-targeted tumors (TU +) and non-magnetically targeted tumors (TU −) were excised and weighed. The radioactivity was measured by a gamma counter (1280 CompuGamma Universal Gamma Counter, LKB Wallac, Finland), using the appropriate energy windows for ^111^In. Results were expressed as percentage of injected dose (% ID) in blood or per gram organ or tumor (% ID/g) as means ± SEM (standard error of the mean) (*n* = 3).

### 2.11 Statistical Analysis

The statistical analysis for each experiment is noted in the figure caption. The One-way ANONA and Tukey’s multiple comparison test were performed for the gamma counting studies, including organ biodistribution, blood circulation and tumor accumulation studies. Student’s t-test was performed for the FCFM quantification studies. All statistical tests were performed using IBM SPSS version 20. The significance (p-value) was determined. When p-values were 0.05 or less, differences were considered statistically significant.

## 3 Results and discussion

### 3.1 Preparation and characterization of magnetic nanocapsules (m-NCs)

We designed a magnetic nanocapsule system (*m*-NCs) encapsulating a high amount of superparamagnetic iron oxide nanoparticles (SPIONs) which enables successful magnetic drug targeting *in vivo*. The m-NCs were designed in such a way that they can be imaged with optical imaging (due to DiR incorporation), MR imaging (due to SPION) and nuclear imaging (by chelation of radioactive indium-111 (^111^In) to diethylene triamine pentaacetic acid (DTPA) conjugated to the polymer). These triple-labeled m-NCs combined with imaging techniques such as SPECT/CT and FCFM allowed us to examine the kinetics of m-NCs accumulation/extravasation in mice organs, including tumors, on a microscopic scale when an external magnetic field was applied.

PEGylated oil-cored m-NCs were prepared using a single emulsification/solvent evaporation method. [16] DiR was encapsulated into the m-NCs for FCFM imaging. m-NCs of increasing SPION loadings: ca. 0-7% w/w SPION/NC were prepared and named as NC 1-5, respectively, were prepared. DiR/SPION encapsulation efficiency (%EE), hydrodynamic sizes (200 to 220 nm) and zeta potentials (− 30 to − 45 mV) are summarized in Table 1 and Table 2.

### 3.2 The correlation between m-NC tumor accumulation and SPION injection dose under passive and magnetic targeting conditions

**Fig. 1:**
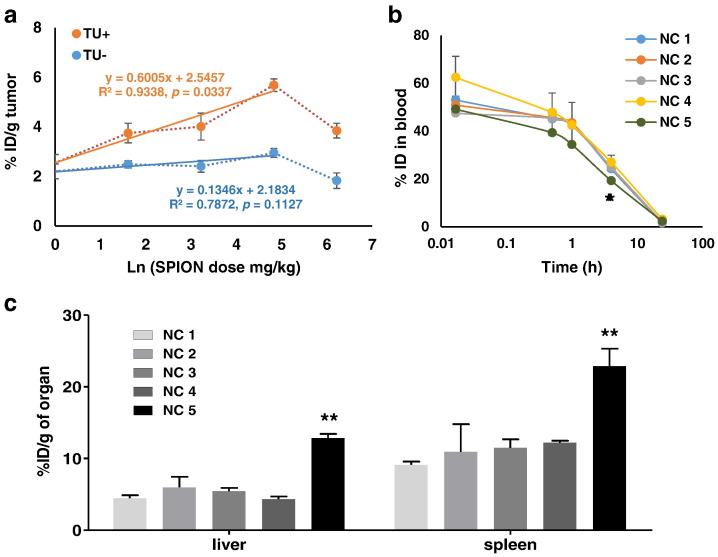
Biodistribution studies of *m*-NC-111In in CT26 tumor-bearing BALB/c mice under the influence of a magnetic field. Mice were intravenously injected with NC-^111^In or *m*-NC-^111^In at SPION content of 0-500 mg/kg. Blood samples were collected at specified time points and organs and tumors were excised at 24 h post-injection. (a) Scatter plots of % ID of *m*-NCs in tumor tissue (24 h) without or with magnetic targeting as a function of natural log of SPION injection dose. The best-fit linear regression line is shown with a correlation coefficient (r2) of 0.9338 in tumor tissue upon magnetic targeting (p = 0.0337). (b) Blood circulation of NC 1-5 with an increasing amount of SPION. (c) Liver and spleen accumulation profiles of NC 1-5 at 24 h post-injection with an increasing amount of SPION. Results are expressed as % ID/g of organ as mean ± SEM (n=3). The linear regression in (a) was performed using Graphpad Prism 5.0. One-way ANOVA was performed using IBM SPSS version 20 followed by Tukey’s multiple comparison test (**p < 0.01).

We firstly assessed the blood circulation time of *m*-NCs with an increasing amount of SPIONs, their uptake in reticuloendothelial system (RES) organs (e.g. liver and spleen), and the tumor accumulation in the presence (TU+) or absence (TU-) of a magnetic field in a subcutaneous CT26 (murine colon carcinoma) tumor model. These *m*-NCs were radio-labeled with ^111^In and their concentrations in blood and tumors were measured by gamma counting. Fig. 1a shows the percentage injection dose (% ID) of *m*-NCs accumulated in the tumor (per gram of tumor) (+/- magnetic field), at 24 h post-injection, against increasing doses of injected SPIONs in *m*-NCs. The tumor accumulation was not affected by increasing the SPION injection dose up to 125 mg/kg (NC 4, ca. 2% w/w) in the absence of a magnetic field (blue line TU-, r2 = 0.7872, p = 0.1127). On the other hand, a linear regression between % ID/g and Ln (SPION dose mg/kg) (red line TU+, r2 = 0.9338, p = 0.0337) was observed from NC 1-4 when a magnetic field was applied. The statistical analysis of the linear regression is provided in Table S1. The good regression between these two parameters indicates the magnetic force was able to influence the tumor uptake and the enhancement of tumor uptake was proportional to the natural logarithm of the injected SPION dose. A significant reduction in tumor uptake was observed when mice were injected with NC 5 with the highest SPION content (ca. 7% w/w, 500 mg/kg). Fig. 1b and c show their blood concentration over time and %ID/g in liver and spleen at 24 h. The high uptake of NC 5 in RES organs corresponded well to the reduced blood circulation time which may lead to its lowered tumor accumulation [18].

### 3.3 Live fibered confocal fluorescence microscopic imaging of m-NCs in tumor blood vessel under the influence of a magnetic field

**Fig. 2:**
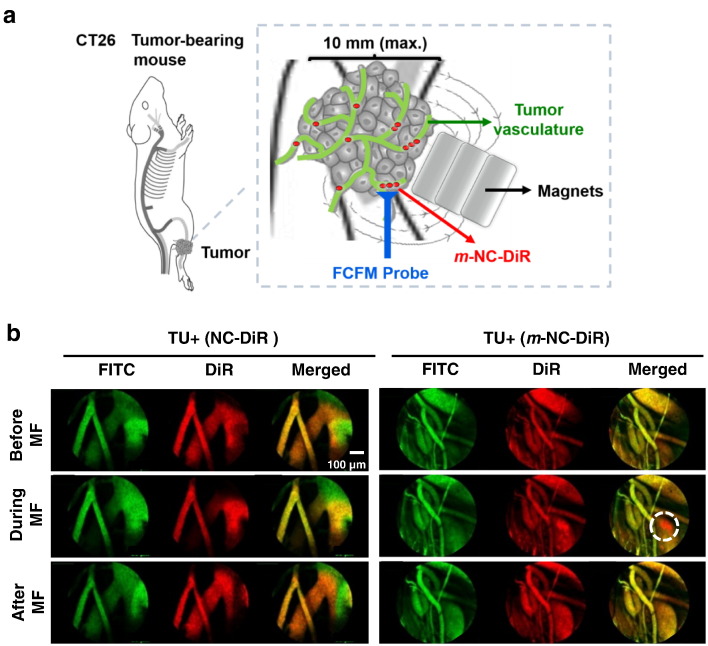
Real time fibered confocal fluorescence microscopic (FCFM) images of magnetic targeting in tumor tissues. Fluorescently labeled non-magnetic NC (NC-DiR) or magnetic NC (*m*-NC-DiR) (red signals) were injected intravenously into CT26 tumor-bearing BALB/c mice and imaged with FCFM using FITC-Dextran (green) as the vascular contrast agent. (a) Schematic illustration of the live FCFM imaging in tumor blood vessels with an external magnetic field (MF) applied at the external part of tumor without direct contact. (b) Representative single frame of live FCFM imaging of NC-DiR and *m*-NC-DiR in tumors before, during and after the application of the magnets. Dashed circle shows the magnetized *m*-NC-DiR, and not NC-DiR is concentrated in the blood vessels upon the application of a magnetic field. All images were acquired using the Cellvizio^®^ dual band imaging system.

The investigation of the effect of magnetic forces on m*-*NC accumulation kinetics in the tumor vasculature is limited by the imaging resolution and invasiveness. As a result, to visualize it dynamically on a microscopic vascular level, a fibered confocal fluorescence microscopy system (FCFM, Cellvizio®, Mauna Kea Technology, Paris, France) was used in this study. Non-magnetic NCs (used as a negative control) and m-NCs were fluorescently labeled with DiR as an optical probe. Fluorescein isothiocyanate–dextran (FITC-Dextran), used as a vascular contrast agent, remained in the blood vessels and no leakage up to 1 h has been reported. Mice were inoculated bi-focally with subcutaneous tumors. The imaging laser probe was inserted into the tumors *via* a small incision and tissues were kept moist throughout the experiment. To dynamically image the travelling of the m-NCs in blood vessels, the FCFM was performed before, during and after the magnetic exposure. The imaging protocol is schematically presented in Fig. 2a.

Prior to application of a magnetic field, DiR-labeled non-magnetic NCs were observed homogenously distributed in blood vessels (Fig. 2b, left panel; Supplementary Movie 1a–c). The application of a magnetic field did not cause any changes in the distribution of non-magnetic NCs within the blood vessels. m*-*NCs, on the other hand, behaved differently when a set of magnets was applied in close proximity to the vessels. DiR labeled m*-*NCs started to magnetize, and those magnetized sufficiently appeared as clusters and enriched near the blood vessel wall (Fig. 2b, right panel; Supplementary Movie 1d–f). The m-NC aggregation was reversible upon removal of the magnetic field due to the superparamagnetism of m*-*NCs [1], [2], [19]. More importantly, the blood vessels appeared intact with no observation of vessel leakage of FITC-Dextran induced by the application of the magnetic field, suggesting no vascular damage *per se*. The enrichment of m-NCs within the tumor microvasculature or further enhanced retention in blood vessels would raise the probability of extravasation into the tumor interstitium and thereby increasing tumor uptake.

**Fig. 3:**
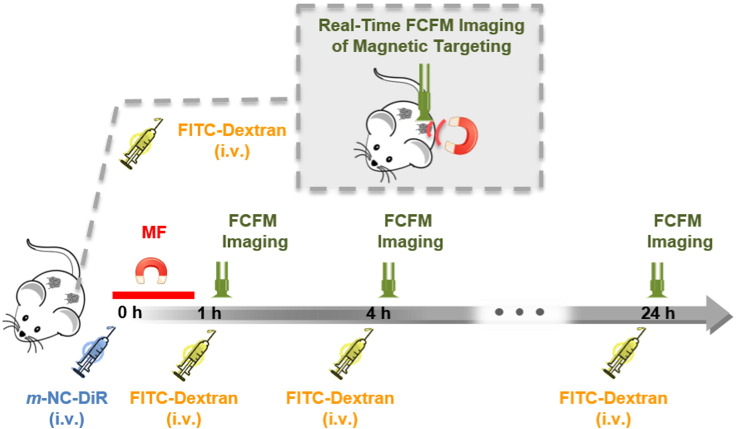
Experimental timeline of fibered confocal fluorescence microscopic (FCFM) imaging of *m*-NCs in tumor vasculature under the influence of a magnetic field (MF). CT26 tumor-bearing BALB/c mice were injected intravenously with m-NC-DiR and a magnet was applied only at one of the bifocal tumors (TU+) for 1 h. FCFM imaging was performed at 1, 4 and 24 h post-injection in both TU+ and TU-. Non-previously injected mouse was used for each time point. FITC-Dextran was injected intravenously at a dose of 500 mg/kg prior to each imaging time point as a macromolecular contrast agent. Inset (top) shows the dynamic FCFM imaging of *m*-NCs in tumor blood vessels during MF application to demonstrate the interaction of *m*-NCs and MF on a microscopic scale.

**Fig. 4:**
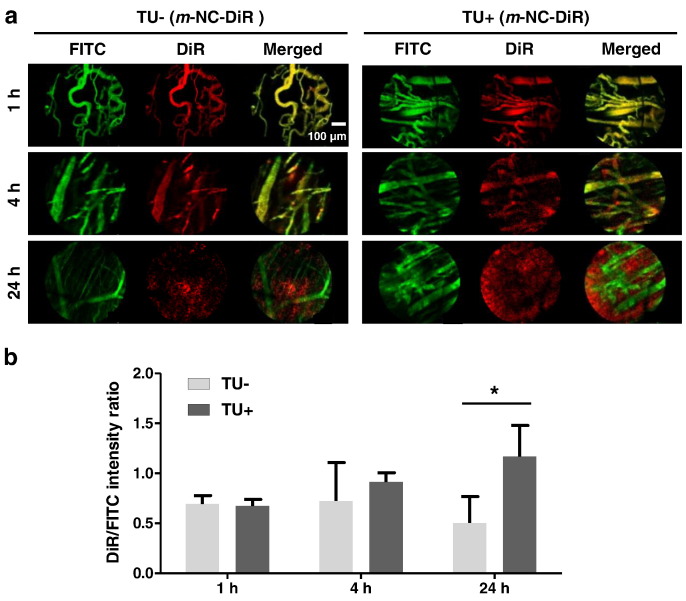
Fibered confocal fluorescence microscopic (FCFM) images of *m*-NCs in tumor tissues over time after the application of the magnetic field. *m*-NC-DiR were injected intravenously into CT26 tumor-bearing BALB/c mice and imaged with FCFM using FITC-Dextran as the vascular contrast agent. A magnetic field was taped at one of tumor surface (TU+) for 1 h and removed afterwards. The contralateral tumor (TU-) was used as a control. (a) Representative single frame FCFM images of *m*-NCs in TU- and TU+ at 1, 4 and 24 h post-injection. (b) Quantification of the fluorescence intensity of *m*-NC-DiR in TU- and TU+. Values are expressed as the ratio of fluorescence intensity from DiR and FITC as mean ± SD (n=3). Paired student t-test was performed using IBM SPSS version 20 (*p < 0.05).

The m*-*NC distribution in both TU − and TU + over time was also studied using FCFM. The multiple imaging schedule is shown in Fig. 3. *m*-NCs were injected at 0 h and a magnetic field was then applied to one tumor for 1 h. Multiple imaging was performed at 1, 4 and 24 h. Tumors are heterogeneous and contain irregular capillary networks and non-vascularized areas of necrosis [20], [21], [22]. FCFM imaging was performed focusing on the highly vascularized regions. FCFM images showed clear co-localization of FITC-Dextran and m-NC-DiR in tumor blood vessels at 1 h and 4 h, confirming the prolonged blood circulation of m*-*NCs (Fig. 4a) [16]. As expected, DiR signals became more diffuse and spread out throughout the tumor interstitium at 24 h. The overall fluorescence intensity of signals from *m*-NC-DiR and FITC-Dextran in TU + and TU - was quantified. The intensity ratios of DiR/FITC were used to compare the accumulation of *m*-NC-DiR in TU+ and TU and the obtained results are shown in Fig. 4b. Interestingly, higher fluorescence intensity of *m*-NC-DiR (higher DiR/FITC ratio) was seen in TU + compared to TU - at 24 h, suggesting enhanced tumor accumulation upon magnetic targeting, consistent with our previously published work [16].

### 3.4 Real-time fibered confocal fluorescence microscopic imaging of m-NCs extravasation in healthy tissues

**Fig. 5:**
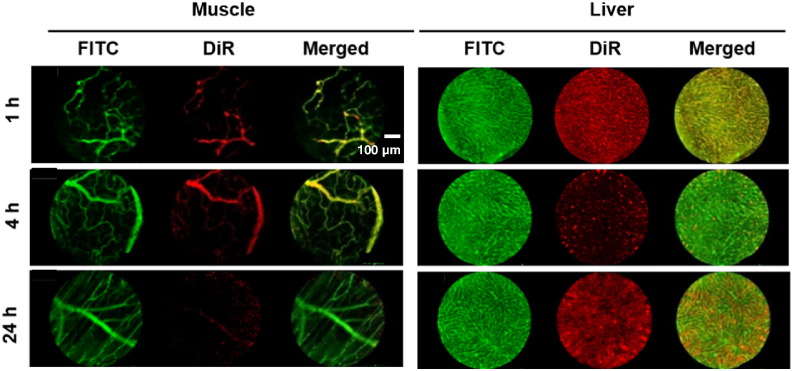
Real-time fibered confocal fluorescence microscopic (FCFM) images of m-NCs in muscle and liver. m-NC-DiR were injected intravenously into CT26 tumor-bearing BALB/c mice and imaged with FCFM using FITC-Dextran as the vascular contrast agent. Representative single frame FCFM images of m-NC in muscle and liver tissues at 1, 4 and 24 h post-injection with the application of a magnetic field at the tumor. All images were acquired using the Cellvizio^®^ dual band imaging system. Scale bar is 50 um.

The extravasation of m-NCs were also assessed in healthy tissues to validate the usefulness for real-time nanoparticle imaging. Muscle and liver which exhibit distinct vascular characteristics were used as examples [23]. Fig. 5 (left panel) shows muscle blood vessels. No DiR signal was visible in the blood vessels of muscle tissues at 24 h, in contrary to the 1 h time point where m-NCs were seen in the vasculature. This is consistent with m-NC distribution data, where no m-NC retention was seen in muscle at 24 h, due to the continuous capillaries and complete basement membrane [16], [24], [25]. It was also confirmed that DiR labelling was confined to the m*-*NCs and the dye did not translocate to cellular membrane. In contrast, a number of m-NCs leaked out of the liver sinusoids and accumulated in non-vascular liver tissues at 24 h (Fig. 5, right panel), consistent with gradual liver accumulation over time described in our previously published work [16]. This was not surprising as the liver vasculature has an incomplete basement membrane and transcytoplasmic openings [25].

It is conceivable from experimental physics and prior knowledge that magnetic nanoparticles (MNPs) can be attracted by the magnetic forces in tumor blood vessel and be concentrated to the tumor tissue. A number of studies indeed have demonstrated that MNPs could be concentrated in the tumor under the influence of a magnetic field by MRI [5], [11]. However, MRI cannot provide sufficient resolution to image the MNP in tumor vasculature. Whether or not the magnetic forces acting on individual m-NC is strong enough to overcome blood flow has not been addressed. A direct visualisation in a time-course study is required.

The FCFM provides real-time *in vivo* microvascular observations and *in situ* high spatial resolution imaging (up to 1.4 μm) at vascular level. It also represents a significant advantage over traditional intra-vital microscopic imaging (IVM) which usually requires invasive surgical procedures and is limited by the availability of animal models that bear visually accessible tumors in a dorsal skinfold chamber. FCFM enables micro-invasiveness and requires only a small incision at the imaging site. Small openings can be sutured if the animals are required to be kept for long-term studies.

## 4 Conclusion

A number of studies have explored magnetic targeting in drug delivery. However, this is the first report of real-time live imaging of magnetic targeting in tumor vasculature on the microscopic scale. The novel application of FCFM described in this study offers direct visualisation of m-NCs within blood vessels and in tumor tissues before, during and after magnetic field exposure in a real-time manner, with high resolution and minimal surgical intervention. Our results suggested that the distribution of m-NCs within tumor vasculature changed dramatically, but in a reversible way, upon application and removal of a magnetic field. The m-NCs were concentrated and stayed as clusters near a blood vessel wall when tumors were exposed to a magnetic field but without rupturing the blood vessel. This work bridges the gap between previous findings of enhanced tumor uptake with magnetic targeting and the nanocarrier behavior at the blood-tumor vasculature-tumor tissue interface, thanks to the high resolution characteristics of this imaging modality. This proof-of-concept descriptive study in mice is envisaged to track and quantify nanoparticles *in vivo* in a non-invasive manner with microscopic resolution.

The following is the supplementary data related to this article.

Supplementary Movie 1 Supplementary Movie 1

## Figures and Tables

**Table 1:** Physicochemical characterization of PEGylated *m*-NCs with different SPION loadings prepared by a single emulsification/solvent evaporation method.

Formulation	Hydrodynamic size (nm)^a^, ^e^	PDI^a^, ^e^	Zeta potential (mV)^b^, ^e^	SPION EE%^c^, ^e^	Final SPION loading^d^, ^e^
NC 1	203 ± 4	0.12 ± 0.01	− 45 ± 2	—	0.00
NC 2	212 ± 2	0.17 ± 0.02	− 38 ± 1	99 ± 3	0.08 ± 0.01
NC 3	218 ± 4	0.17 ± 0.02	− 39 ± 1	99 ± 2	0.38 ± 0.01
NC 4	205 ± 3	0.16 ± 0.01	− 36 ± 1	95 ± 3	1.76 ± 0.06
NC 5	214 ± 9	0.19 ± 0.02	− 31 ± 1	94 ± 9	6.60 ± 0.63

a Size was measured with dynamic light scattering and measured in deionized water

b Values were obtained with laser Doppler electrophoresis and measured in deionized water

c Iron content was determined by ICP-MS

d Weight of NCs includes polymer, lecithin, castor oil, SPION and tween 80^®.^ Initial and final loading refers to SPION content before and after size exclusion chromatography.

e Results are expressed as mean ± SD (n = 3)

**Table 2:** Physicochemical characterization of PEGylated DiR encapsulated NCs and *m*-NCs prepared by a single emulsification/solvent evaporation method.

Formulation[a]	Hydrodynamic size (nm)^b^, ^e^	PDI b, e	Zeta potential (mV)^c^, ^e^	DiR EE%^d^, ^e^
NC-DiR	206 ± 5	0.16 ± 0.01	-39 ± 2	99 ± 4
*m*-NC-DiR	209 ± 2	0.19 ± 0.02	-32 ± 1	95 ± 5

a Formulations were prepared based on NC 4

b Size was measured with dynamic light scattering and measured in deionized water

c Values were obtained with laser Doppler electrophoresis and measured in deionized water

d Values were determined by fluorescence spectroscopy

e Results are expressed as mean ± SD (n = 3)
