# Supplementary figures and images for: Real-time monitoring of magnetic drug targeting using fibered confocal fluorescence microscopy
Source: J Control Release. 2016 Dec 28;244(Pt B):240–6. doi: 10.1016/j.jconrel.2016.07.026 (PMC5176088; doi:10.1016/j.jconrel.2016.07.026)

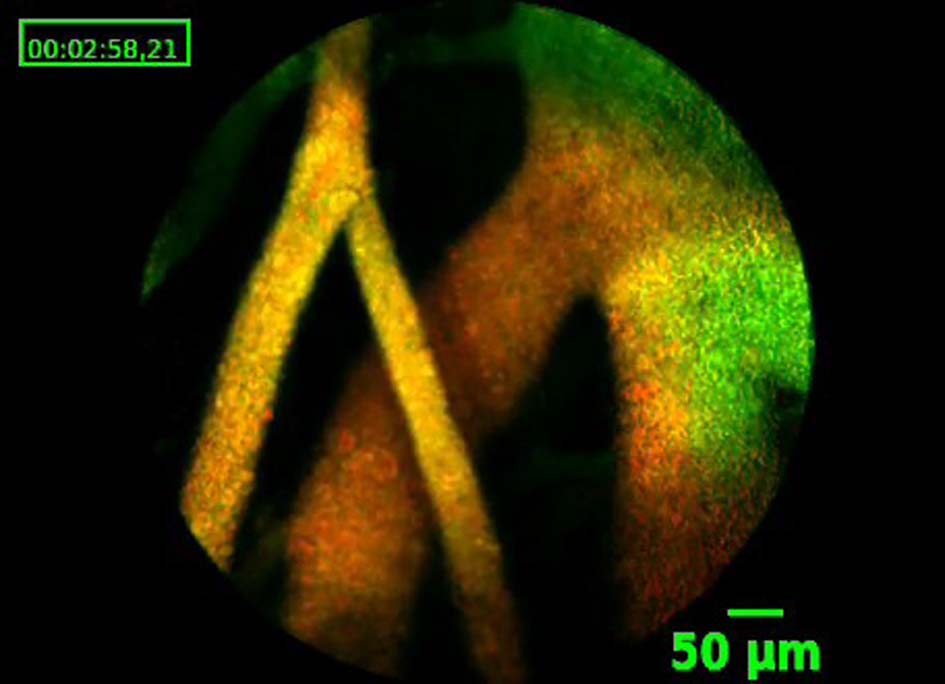

Supplement: Supplementary Movie 1 [file mmc1.jpg]

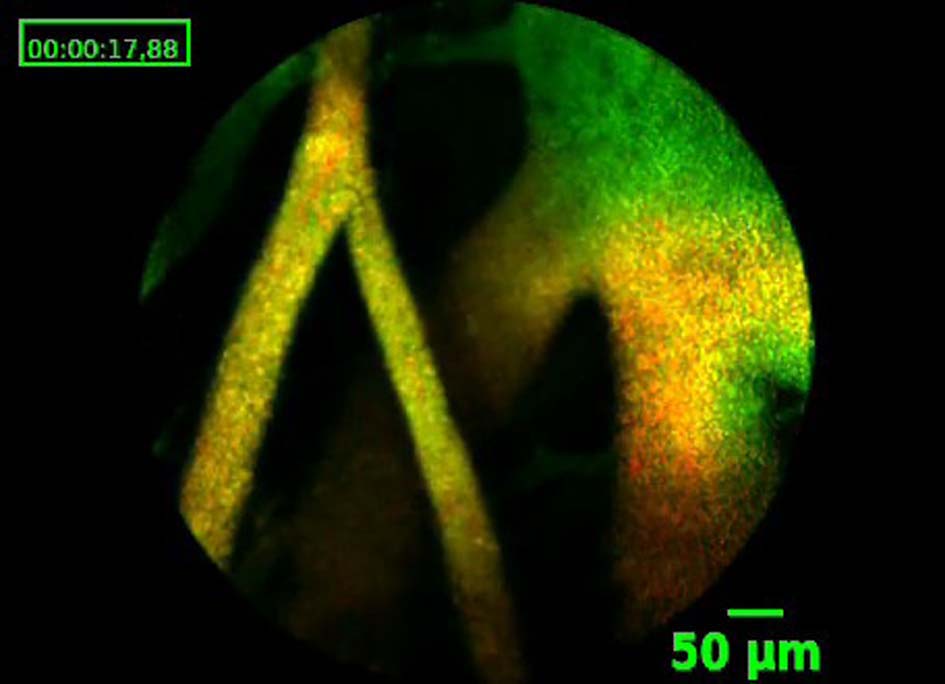

Supplement: Supplementary Movie 1 [file mmc2.jpg]

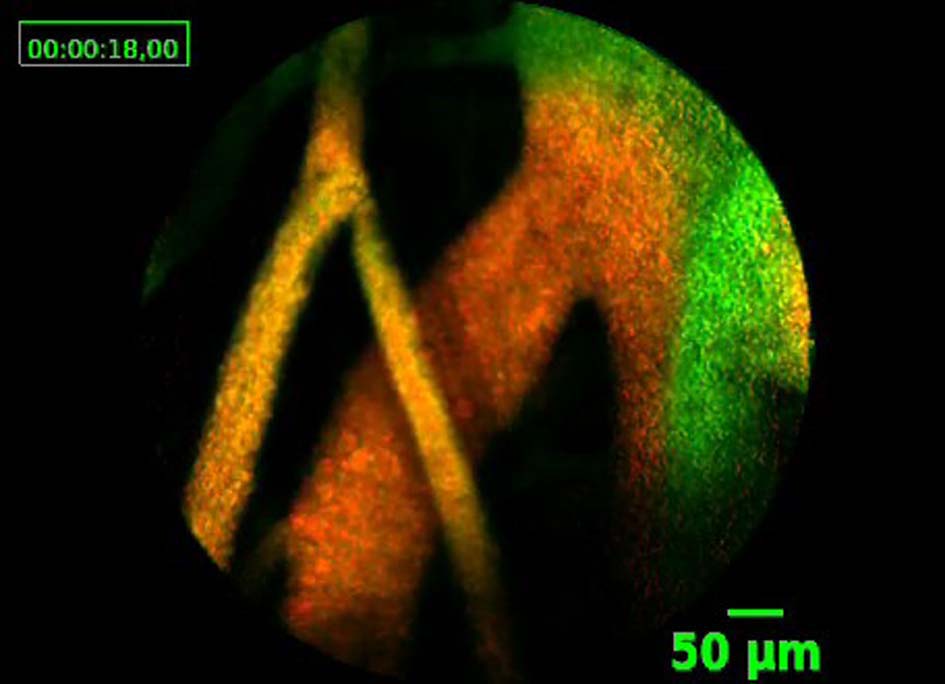

Supplement: Supplementary Movie 1 [file mmc3.jpg]

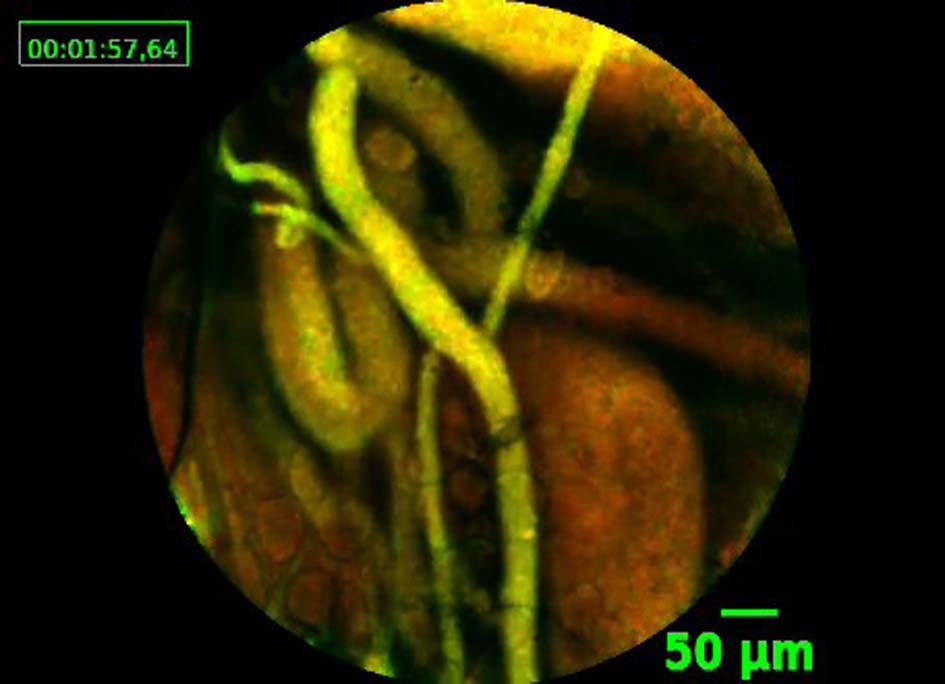

Supplement: Supplementary Movie 1 [file mmc4.jpg]

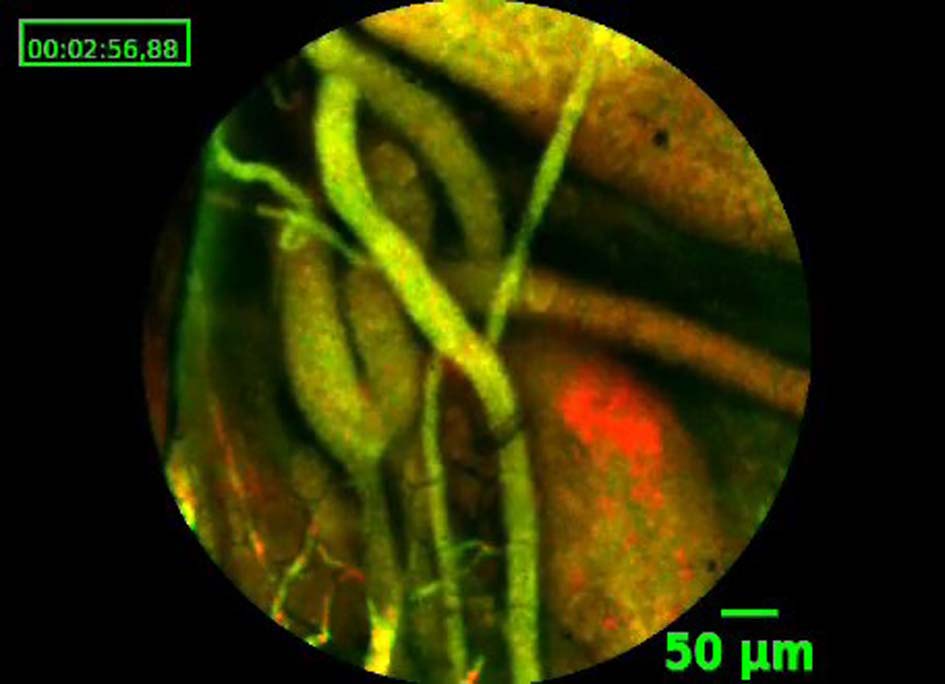

Supplement: Supplementary Movie 1 [file mmc5.jpg]

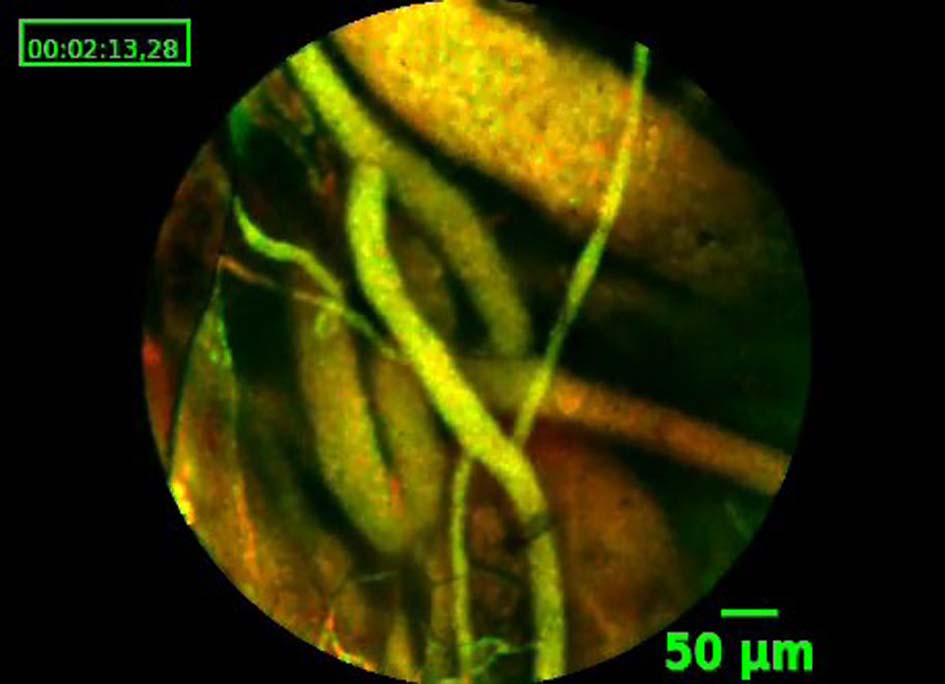

Supplement: Supplementary Movie 1 [file mmc6.jpg]
